# Will AI Replace Ophthalmologists?

**DOI:** 10.1167/tvst.9.2.2

**Published:** 2020-01-29

**Authors:** Edward Korot, Siegfried K. Wagner, Livia Faes, Xiaoxuan Liu, Josef Huemer, Daniel Ferraz, Pearse A. Keane, Konstantinos Balaskas

**Affiliations:** 1 NIHR Biomedical Research Center at Moorfields Eye Hospital NHS Foundation Trust and UCL Institute of Ophthalmology, London, UK; 2 Department of Ophthalmology, Cantonal Hospital Lucerne, Lucerne, Switzerland; 3 Department of Ophthalmology, University Hospitals Birmingham NHS Foundation Trust, Birmingham, UK; 4 Academic Unit of Ophthalmology, Institute of Inflammation & Ageing, University of Birmingham, Birmingham, UK; 5 School of Biological Sciences, University of Manchester, Manchester, UK

**Keywords:** artificial intelligence, AI, algorithms, future

## Introduction

Teamwork, creativity, adaptability, empathy—all traits that physicians employ on a daily basis to effectively deliver patient care. One may argue that these are elements of physician-patient interaction that artificial intelligence (AI) could never replicate. However, others would contend that AI models have already demonstrated some of these features. Recent notable examples include AI mastering cooperative gameplay and generative adversarial networks creating novel artwork and melodic music.^1^^–^^4^ These advances were all made possible due to the recent proliferation of deep neural networks, which have also ushered a stepwise improvement in machine learning performance in ophthalmology.^5^^–^^8^ However, it is crucial to clarify that these and similar AI models that show creativity, teamwork, and adaptability are examples of “narrow” AI. These algorithms are typically validated in constrained testing environments and have limited generalizability. Furthermore, when evaluated outside their test environments in a more abstract fashion or presented with intentional adversarial counterfactuals, they often fail with unfortunate consequences.^9^

## AI Challenges

Even considering the aforementioned examples, which mimic certain elements of human behavior, there has not yet been a demonstration of empathy by an AI algorithm. In the context of medicine, empathy comprises not only understanding a patient's feelings but, more important, also responding by delivering care in an appropriate manner. A health professional's relationship with the patient helps guide the patient's care in the context of his or her unique physical, emotional, and social environment. Furthermore, the doctor-patient relationship itself has been shown to have a therapeutic effect in a systematic review of 25 randomized controlled trials.^10^ The patient-clinician interaction is innately human and, in the words of patients themselves, depends on “two humans who both can fully contextualise and appreciate the patient's values, wishes, and preferences.”^11^

**Figure 1.**
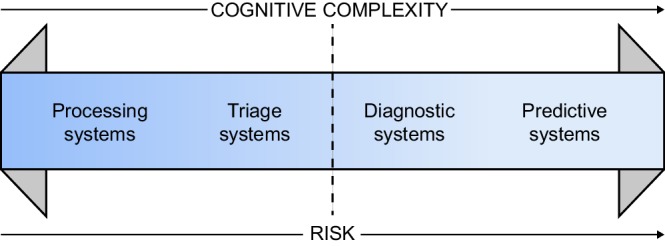
Risks of medical AI. AI model risk increases as cognitive complexity increases. We currently lie at the dotted line, a tipping point between triage and diagnostic systems. Permission received for reproduction from Luke Oakden Rayner.^16^

Beyond the human interaction component, translating AI from laboratory experiment to a real-world tool entails additional challenges. “Do no harm,” the first line of the Hippocratic Oath, signifies that physicians employing tools such as AI in patient care delivery must maintain safety as the first priority. As Luke Oakden-Rayner,^12^ a radiologist and critical AI blogger explains, Silicon Valley's ethos of “move fast break things” can be especially dangerous in the context of medical AI. When AI-assisted medical care transitions from triage to diagnostic systems (Fig. 1), so too the inherent risk increases. In ophthalmology, we currently lie at the “dotted line,” as triage systems such as the ones developed by IDX and Google DeepMind are precursors of future diagnostic and predictive systems.^5^^,^^13^ The Moorfields DeepMind algorithm already has a diagnostic component, and predictive systems are just around the corner. Arcadu et al.^14^ describe a system capable of predicting two-step worsening of diabetic patients’ Early Treatment of Diabetic Retinopathy Study (ETDRS) scores at 12 months with an area under the receiver operating characteristic curve (AUC) of 0.79, and a Google group recently described an algorithm that could predict acute kidney injury 48 hours in advance.^15^

As these AI systems are poised to influence clinical decision making, the risks become more apparent, and prospective validation becomes more important. As Oakden-Rayner^12^ suggests, validation studies should focus on clinical outcomes of AI system implementation and not simply on prospective algorithm performance validation. Ophthalmologists need to aim for patient-specific outcomes such as vision loss and disability while maintaining focus on health care–specific outcomes such as reducing money spent per intravitreal injection or surgery.^16^^,^^17^ Laboratory performance does not equal outcomes, and this was highlighted by the case of mass adoption of computer-aided diagnosis (CAD) for mammography screening. Although early reader studies showed that computers working with radiologists led to better accuracy than radiologists alone, subsequent clinical trials demonstrated that false-positive rates increased after CAD adoption. This led to an almost 20% increase in the rate of biopsies, confirming the potential disconnect between diagnostic accuracy and clinical effectiveness.^16^^,^^17^

Furthermore, as predictive algorithms become implemented in clinical decision making, false positives may become a self-fulfilling prophecy.^18^ An ophthalmologist will not know whether the patient predicted to develop proliferative diabetic retinopathy, who was subsequently treated with a prophylactic anti-Vascular endothelial growth factor (VEGF) injection, will have ever developed the disease if he or she was not treated. When used in this manner, the algorithm's false-positive rates would be extremely difficult to detect—even in large outcomes-based clinical trials. If that so-called false-positive patient then went on to develop an iatrogenic complication, unnecessary harm would be inflicted and would be difficult to attribute to the AI's recommendation in any post hoc analyses.

## Limitations of Deep Learning in Ophthalmology

Even with the success of deep learning–based AI models in the research setting, we must be cautious by not inferring similar performance in real-world use. Although deep learning has led to substantial advances in image classification, it is not without shortcomings. Gary Marcus^19^ has well summarized the limitations; specific criticisms that are relevant to ophthalmology include insufficient transparency, poor integration with prior hierarchical knowledge, and inflexibility. Furthermore, a model's evaluation metric may not be indicative of the product's or patient's clinical goals.^20^ In an illustrative clinical scenario, a deep learning AI model that was optimized for reducing macular thickness may prove irrelevant, as evidence becomes available that visual acuity may not correlate with this goal. This inflexibility and inability to encode hierarchical prior knowledge could quickly lead to a model's gross underperformance and rapid obsolescence.

AI models may break down when they encounter dissimilar image acquisition and patient-specific variables from those the model was trained on. Although prospective observational clinical validation is crucial to ensuring real-world model performance, this kind of data is often lacking.^21^ It is frequently difficult to determine the precise reason that these models fail, and they are therefore often termed “black boxes.”^20^^,^^22^^,^^23^ Furthermore, if these models fail in screening settings, which often affect larger numbers of people, unnecessary additional interventions have the potential for even higher impact. This was exemplified by post hoc analyses of prior large-scale computer-aided screening programs.^17^^,^^24^

## Novel Uses of Ophthalmic AI

Nevertheless, advances in AI have not only shown levels of performance that may supersede human ophthalmologists but have also demonstrated proficiency in tasks that were not previously thought possible for ophthalmologists to perform. Perhaps the most striking is a recent demonstration that a deep learning algorithm could accurately predict cardiovascular risk factors and demographics from fundus photos.^4^^,^^25^ This AI model predicted age with a mean absolute error (MAE) of 3.26 years, sex with an AUC of 0.97, smoking status with an AUC of 0.71, and systolic blood pressure with a MAE of 11.23 mm Hg. Although the ophthalmoscope was invented in the mid-1800s, and ophthalmologists have been looking at the fundus for as many years, these insights were not previously conceivable.

Recent evidence continues to support the view that the eye is a window to the vascular and central nervous systems of the body. Associations between retinal findings and neurodegenerative and cardiovascular diseases such as Alzheimer disease and hypertension have been increasingly validated.^26^^–^^28^ Future advances in AI-based ophthalmic image analysis will undoubtedly demonstrate unforeseen disease associations and their ophthalmic correlates. The ability of AI systems to detect pixel-level patterns among millions of pixels per image, comprised in data sets approaching millions of patients, will never be matched by ophthalmologists. These occult patterns may enable not only earlier systemic disease detection but also novel insights into the pathophysiology of ophthalmic and systemic diseases.

## The Potential of an Ophthalmologist-AI Partnership

Effective clinical medicine and ophthalmology with its large data sets of longitudinal imaging will ultimately benefit from collaborating with AI. Verghese et al.^29^ describe humans working with machines and emphasize the lead time that predictive models can offer for diagnosis and action. However, these models can only lead to effective clinical decisions if they keep human intelligence “in the loop” to bring context. As with other imaging-heavy specialties such as radiology, ophthalmology is positioned to lead the uptake of medical AI. However, unlike radiologists, ophthalmologists additionally employ specialized examination skills and perform complex microsurgery. Therefore, ophthalmology is both uniquely positioned to take advantage of AI yet also uniquely protected against obsolescence to machines.

Concerns about physician unemployment have historically been raised with any stepwise improvements in automation and are often out of proportion to reality. Verghese et al.^29^ reference an editorial from 1981 on using predictive risk factors from a then-novel computer database, stating that “proper interpretation and use of computerized data will depend as much on wise doctors as any other source of data in the past.”^30^ While a recent US report states 47% of jobs are at risk for automation, the risk for physicians and surgeons is estimated to be 0.4%.^31^^,^^32^

As various automations in medicine have freed clinicians from menial tasks, AI will continue that trend by integrating the ever increasing volumes of clinical, genomic, and imaging data. This will allow the ophthalmologist to focus on providing effective and compassionate clinical care. Currently, the majority of time is spent collating and synthesizing data and a minority interacting with the patient. However, with such vast volumes of data, the clinician would in effect be forced to use an aid to perform the data processing and thus have more time to be “deeply human” with the patient.

One can imagine a “clinic of the future”—a term described by Eric Topol,^33^ in which a patient presents with multimodal high-resolution images, functional testing, genomic sequencing, metabolomic/proteomic information, and sensor data from home monitoring. Ophthalmologists would be provided a concise summary comprising structural and functional trends. They may also have access to richly annotated imaging segmented and highlighted for trending changes. Additional AI-synthesized predicted images could be presented of disease course depending on various treatment regimens. Consequently, ophthalmologists would then use this information as an additional tool and, together with the patient, formulate a treatment plan. They would subsequently compare this plan with the AI's recommendations, as well as its predictions of disease course and treatment response to select the best course of action based on the patient's unique circumstances.

## Humans (and Human Ophthalmologists) Are Underrated

Time is a precious commodity for both patients and ophthalmologists. Patients often complain of insufficient doctor contact; similarly, physicians are increasingly burnt out from more time spent on clerical tasks than patient care.^34^ Although new technology often promises efficiency improvements, as with the case of the electronic health records, one can see that such promises frequently fail to deliver. If implemented correctly, AI is unique in its potential to save time by processing large longitudinal data volumes and efficiently representing the patterns identified. Ophthalmologists will have more time for physical patient contact—everting an eyelid to discover a hidden conjunctival melanoma, performing a thorough gonioscopy or cranial nerve examination, or perfecting their surgical technique.

As described by Geoff Colvin,^35^ human brains were designed for social interaction. No patient would want to be informed that they have a terminal disease or that they are going blind by their AI assistant. The new high-value skills will become those that “literally define us as humans,” sensing the thoughts and feelings of patients losing vision, coordinating assistive devices with family members, and allowing the patients to express themselves about how their eyesight affects their lives. Although many ophthalmologists disagree with the concept of patient satisfaction influencing reimbursement, this relatively new development is an example of the increasing value being placed on such human-metrics. Colvin^35^ states, “It used to be that you had to be good at being machinelike. Now, increasingly, you have to be good at being a person. Great performance requires us to be intensely human beings.”

## Conclusion

We currently lie in a stage between AI demonstration and deployment. Next comes ongoing evaluation, learning, model adjustment, and finally meaningful human-AI interaction. Ophthalmologists should leverage the primary strength of AI, its ability to glean insights from large volumes of multivariate data, with their abilities to interpret the AI's recommendations in a clinical and societal context. In doing so, the field will be well positioned to lead the transformation of health care in a positive and personalized direction. As more time will become available for human-suited tasks, ophthalmologists will have more time to be human—we will just use a digital helping hand from AI.
